# Prefrontal Cortex Haemodynamics and Affective Responses during Exercise: A Multi-Channel Near Infrared Spectroscopy Study

**DOI:** 10.1371/journal.pone.0095924

**Published:** 2014-05-01

**Authors:** Gavin D. Tempest, Roger G. Eston, Gaynor Parfitt

**Affiliations:** 1 School of Health Sciences, The Sansom Institute for Health Research, University of South Australia, Adelaide, SA, Australia; 2 Sport and Health Sciences, University of Exeter, Devon, United Kingdom; University of Missouri-Kansas City, United States of America

## Abstract

The dose-response effects of the intensity of exercise upon the potential regulation (through top-down processes) of affective (pleasure-displeasure) responses in the prefrontal cortex during an incremental exercise protocol have not been explored. This study examined the functional capacity of the prefrontal cortex (reflected by haemodynamics using near infrared spectroscopy) and affective responses during exercise at different intensities. Participants completed an incremental cycling exercise test to exhaustion. Changes (Δ) in oxygenation (O_2_Hb), deoxygenation (HHb), blood volume (tHb) and haemoglobin difference (HbDiff) were measured from bilateral dorsal and ventral prefrontal areas. Affective responses were measured every minute during exercise. Data were extracted at intensities standardised to: below ventilatory threshold, at ventilatory threshold, respiratory compensation point and the end of exercise. During exercise at intensities from ventilatory threshold to respiratory compensation point, ΔO_2_Hb, ΔHbDiff and ΔtHb were greater in mostly ventral than dorsal regions. From the respiratory compensation point to the end of exercise, ΔO_2_Hb remained stable and ΔHbDiff declined in dorsal regions. As the intensity increased above the ventilatory threshold, inverse associations between affective responses and oxygenation in (a) all regions of the left hemisphere and (b) lateral (dorsal and ventral) regions followed by the midline (ventral) region in the right hemisphere were observed. Differential activation patterns occur within the prefrontal cortex and are associated with affective responses during cycling exercise.

## Introduction

A dose-response relationship exists between the intensity of exercise and the pleasure-displeasure individuals feel [1], [2]. These affective responses, which underlie core affect [3], are linked to future exercise participation [4]–[6]. Recent guidelines from the American College of Sports Medicine (2013) recognise the importance of affective responses in exercise prescription and the detrimental impact of negative affect on future exercise participation [7]. This recognition provides the rationale to investigate a proposed neural basis of affective responses during exercise, particularly the role of the prefrontal cortex (PFC).

The dose-response relationship between the intensity of exercise and affective responses emerged following recommendations to standardise exercise intensities to individual gas exchange thresholds; the ventilatory threshold (VT) and the respiratory compensation point (RCP) [8]. The VT (approximately 60–70% peak oxygen uptake [VO_2peak_]) indicates the point of transition from aerobic to anaerobic metabolism and the RCP (approximately 80–90% VO_2peak_) indicates the point at which a physiological steady state can no longer be maintained [9]. At intensities of exercise substantially below VT, individuals report mostly positive (pleasant) affective responses [10], [11]. However at intensities above VT (proximal to RCP and exhaustion), individuals report uniformly negative (unpleasant) affective responses [12]–[15].

The dose-response relationship is explained by a theoretical framework, called the dual-mode model [16], [17]. According to the model, affective responses to exercise are regulated by the PFC and subcortical areas of the brain (which receive sensory input from the body). It is proposed that activation in the PFC serves to maintain positive (or actively avoid negative) affective responses during exercise. However, as the intensity increases (i.e. above VT) the functional capacity of the PFC becomes challenged by the intensified sensory (interoceptive) input from the body. Therefore, a deregulation in the PFC is proposed to occur, resulting in a negative-affective response, mainly driven by subcortical structures (i.e. the amygdala). Deregulation of the PFC would allow a shift of metabolic resources to areas of the brain directly associated with the activity [18], [19]. The basis of the dual-mode model is that the PFC provides top-down regulation (i.e. inhibition of subcortical structures) of affective responses through cognitive control processes. However at intensities of exercise above VT, deregulation of the PFC may occur and the functional capacity of the PFC becomes limited.

The PFC is involved in executive function and cognitive control processes [20]. *Cognitive control* describes an individual’s ability to facilitate or inhibit stimuli at a neural level, which may influence their behavioural (and affective) response [21]. Two main outcomes of affective (and emotional) regulation are (a) inhibition or suppression of stimuli through attentional processes, which serve to focus towards or distract away from stimuli and limit expressive action, and (b) cognitive reappraisal (defined by Lazarus [22]), which involves manipulating the meaning of stimuli in relation to current goals which neutralise negative experiences and therefore influence the on-going emotional response [21]. Evidence from neuroimaging studies has indicated differential activation patterns in areas of the PFC during cognitive control tasks in the presence of negative affective stimuli. For example, activation in the dorsolateral and ventromedial PFC is increased and is associated with reduced self-reported negative affect [23]. Activation in the right dorsolateral PFC is increased in attempts to inhibit [24] and supress [20], [25], emotional responses. Cognitive control has been shown to induce greater activation in left-lateralised areas of the PFC in the presence of positive stimuli [26], [27], and greater activation in right-lateralised areas of the PFC in the presence of negative stimuli [27]. Further, neuroimaging studies have shown that activity in areas of the PFC induced by attempts of active cognitive coping with a negative affective stimulus, are associated with reduced activity in the amygdala [26]–[30].

Prefrontal lateralisation of affective (positive-negative) processing is related to the approach-withdrawal motivational system described by the valence asymmetry hypothesis [31], [32]. The approach system is associated with positive affect, which drives goal-orientation and behaviours towards something [22]. In opposition, the withdrawal system is associated with negative affect and aversion, which drives behaviours away from something. Studies have provided neurophysiological evidence of increased left anterior activation and approach-related positive affect and increased right anterior activation and withdrawal-related negative affect within the frontal cortex using electroencephalography [33]–[35], functional magnetic resonance imaging [36] and near infrared spectroscopy (NIRS) [37]. In exercise studies, an association between frontal cortex asymmetry and affective responses has previously been reported [38]–[40]. However, in line with the dual-mode model, the functional capacity of the PFC to regulate affective responses, presumably through cognitive control (i.e. appraisal, inhibition and/or suppression) and lateralised processes during exercise intensities relative to the VT and RCP has not been explored.

Advances in NIRS allow measures of cerebral haemodynamics to be taken during exercise. Optodes (light emitters and detectors) placed over the cortex indicate the change (Δ) in near-infrared light (due to absorption) as it passes through the underlying tissue and provides a measure of oxy- (O_2_Hb) and deoxy-haemoglobin (HHb) concentration [41]. Cerebral ΔO_2_Hb and ΔHHb reflects functional neuronal activation [42], [43]. Several studies have shown that cerebral oxygenation (ΔO_2_Hb) in frontal areas is increased during sub-maximal exercise however it is reduced during exercise at near maximal exercise intensities [42], [44]–[47]. The interpretation of these findings is that a reduced supply of oxygen in frontal areas limits frontal cortex activation and contributes to the cessation of exercise. A meta-analysis by Rooks, Thom, McCully & Dishman [48] summarised the cerebral haemodynamic response during incremental exercise. The authors showed that at sub-maximal intensities (≥60% VO_2peak_; around the VT) increases in cerebral oxygenation (ΔO_2_Hb; blood flow), deoxygenation (ΔHHb) and blood volume (total haemoglobin [ΔtHb]; the sum of ΔO_2_Hb and ΔHHb) were observed. As exercise increased towards maximal intensities (VO_2peak_) cerebral oxygenation (ΔO_2_Hb) and blood volume (ΔtHb) were lower, with a continued rise in deoxygenation (ΔHHb). These findings indicate that a reduced supply of oxygen (ΔO_2_Hb) compared to relative demand (ΔHHb) occurs during exercise, therefore PFC activation is limited. Measures of cerebral haemodynamics using NIRS during incremental exercise are generally limited to single (as opposed to bilateral or multiple-) areas of the PFC [41], [48]. However, multi-channel NIRS has been used to examine cerebral haemodynamics from frontal and motor areas of the cortex, which have shown greater deoxygenation in frontal compared to motor areas at near maximal exercise intensities [49]. Yet no comparison of NIRS measures recorded from multiple areas (dorsal and ventral) of the PFC during incremental exercise is available. Ekkekakis [50] proposed that variations in the haemodynamic response of the PFC may represent individualised neuronal activity. Therefore measures of the haemodynamic response from multiple areas of the PFC could potentially indicate functional activation (i.e. top-down regulatory [cognitive control] processes) due to affective processing.

According to the dual-mode model, a reduction in oxygenation (ΔO_2_Hb) in the presence of increased deoxygenation (ΔHHb) might indicate reduced neuronal activation and therefore a reduced functional capacity of the PFC, which may influence the onset of negative affective responses. Consistent with the dual-mode model, the transient ‘hypofrontality’ hypothesis [18], [19], states that the capacity of the PFC to maintain cognitive and affective processes is challenged during exercise due to a redistribution of metabolic resources from cortical to subcortical areas of the brain. During exercise, global cerebral blood volume remains relatively constant. However there is a shift in resources (oxygen delivery, metabolite removal) [44] or greater oxygen consumption [49] between areas required for cognitive, emotional, sensory and motor control. Regional activation in the brain comes at the expense of other regions [20]. Dietrich [18], [19], describes a five-stage hierarchy of consciousness of which the first to ‘deregulate’ are higher-order processes (in the dorsolateral PFC); the second, self-representation and emotional processes (in the ventromedial PFC); the third, memory, learning and basic emotions (the limbic system including the amygdala); the fourth, sensory processing (in the thalamus); and finally, arousal processes (in the brainstem). It is proposed that affective responses are largely influenced by the functional capacity of the PFC to maintain activation in competition with sensory (interoceptive) inputs from the body during exercise as the intensity becomes increasingly challenging.

Due to the limited depth resolution of NIRS only activation from surface regions of the cortex can be measured. No measures of activation in subcortical areas of the brain (i.e. amygdala and thalamus) can be recorded. To the authors’ current knowledge, haemodynamics in regions of the lateral PFC, such as dorsal and ventral areas, using multi-channel NIRS during incremental exercise has not been examined. In line with the dual-mode model and evidence of cognitive control and lateralised affective processes of the PFC, it is proposed that differential activation patterns would occur as the intensity of exercise increases. Differential activation of the PFC is theorised to coincide with reports of negative affective responses. It is further proposed that variability in the haemodynamic response highlighted by Rooks et al. [48], could be attributed to measures recorded from multiple regions of the PFC (as opposed to single uni- or bilateral measures not considered by Rooks et al.) during exercise standardised to metabolic markers.

Therefore, the present study investigates the functional capacity (activation in sub regions) of the PFC (reflected by haemodynamics using multi-channel NIRS) and affective responses during exercise at intensities standardised to below VT, VT, RCP and the end of exercise (recommended by Ekkekakis [50] and Rooks et al. [48]).

Three hypotheses were examined: 1) activation in the PFC is different in sub regions (dorsal and ventral area) at intensities of exercise above the VT; 2) affective responses are more positive at exercise intensities corresponding to below VT and more negative at exercise intensities corresponding to RCP and the end of exercise; and 3) activation in sub regions of the PFC is associated with affective responses during exercise.

## Methods

### Ethics Statement

This study was approved by The University of South Australia Ethics Committee. Participants read and signed an informed consent form prior to participation in the study.

### Participants

Healthy participants (*n = *25; male = 13; mean ± SD; age 25.6±3.4 years; height 174.8±6.8 cm; body mass 71.8±8.3 kg; body mass index 23.5±2.2; VO_2peak_ 41.8±5.2 ml·kg^−1^·min^−1^) were recruited. The volunteers completed the Adult Pre-exercise Screening System (APSS [51]) prior to the study to ensure suitability for participation. All participants were non-smokers and recreationally active. Participants reported partaking >3 hours of moderate physical activity per week determined by the APSS.

### Measures

#### Near infrared spectroscopy

**Figure 1:**
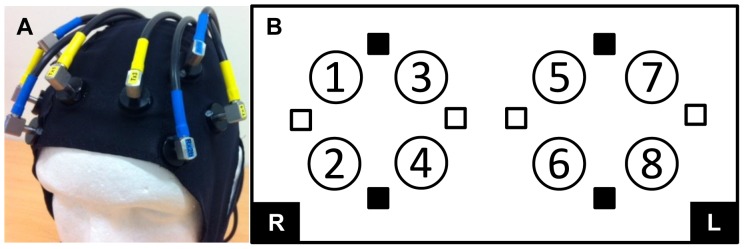
Optode arrangement. (A) Photograph of the optode cap, and (B) diagram of the optode arrangement (emitters □ and detectors ▪) over the right (R) and left (L) prefrontal areas of the cortex. The numbers (1–8) represent the sub regions measured. Odd numbers identify dorsal and even numbers identify ventral regions.

Cerebral haemodynamics were measured in eight sub regions (1–8) of the PFC using multi-channel NIRS (Oxymon Mk II, Artinis Medical Systems, Zetten, the Netherlands). Optodes (four emitters and four detectors) were secured into optode holders on a head cap. The optode holders were arranged in two squares (four holders 38 mm equidistance apart). The four optodes, two emitters and two detectors of each square measured four regions over the left and right dorsal and ventral areas of the PFC. The emitter and detector optodes were arranged as shown in Figure 1. The dorsal regions (1, 3, 5, 7) were approximately placed over Brodmann’s areas (BA) 45 and 9/46 and ventral regions (2, 4, 6, 8) were approximately placed over BA 10 and 47 using the 10/20 international standardised system for electrode placement. Once the cap was fitted to each participant the placement and distance between the optodes was adjusted and secured. A black bandage was secured over the top of the optodes and around the head to shield from any extraneous light. Age-specific differential path-length factors [4.99+0.067 (age^0.814^)] were calculated [52] using the modified Beer-Lambert equation to provide a measure (sample rate 2 Hz) of the concentration changes (micromolar; µM) in ΔO_2_Hb, ΔHHb ΔtHb and haemoglobin difference (ΔHbDiff; ΔO_2_Hb − ΔHHb). Cerebral ΔHbDiff represents oxygen supply (saturation) versus demand (extraction) and reflects activation.

#### Affective responses

Affective valence (pleasure-displeasure) was measured using the Feeling Scale [53]. This uni-dimensional 11-point scale (ranging from −5 to 5 with verbal anchors at all odd integers, and at zero point; −5 very bad, −3 bad, −1 fairly bad, 0 neutral, 1 fairly good, 3 good, 5 very good) allows multiple assessments to be made during exercise (i.e. pre-, during and post-exercise) to capture any acute fluctuations in responses. The Feeling Scale corresponds to one of the two dimensions of the circumplex model of feeling states [3] and is recommended to measure basic affect (pleasure-displeasure) [1], [2], [8].

### Procedures

The study required individuals to visit the exercise physiology laboratory (temperature ∼24°C, and relative humidity ∼40%) on one occasion. Upon arrival, participants completed the informed consent form and exercise testing questionnaire and initial assessments were taken (age, height and weight). The procedures for the exercise tests were explained and a description of the Feeling Scale was provided. Participants were seated on a recumbent cycle ergometer (Lode Corival, Groningen, the Netherlands) and the NIRS optodes were carefully positioned. A facemask was fitted to measure metabolic data via online gas analysis (Cortex Metalyzer 3B, Biophysik, Leipzig, Germany). The participants then completed an incremental (20 Watts/min; pedal cadence 70 rpm) cycling exercise test to exhaustion. The end of the test was determined by volitional cessation of exercise or failure to maintain a pedal cadence of 70 rpm despite strong verbal encouragement. Cerebral haemodynamic responses and expired gases were measured continuously and affective responses were recorded pre-, during (every minute) and at the end of exercise (VO_2peak_). The achievement of VO_2peak_ was verified by a) a peak or plateau in oxygen consumption (changes <2 ml·kg^−1^·min^−1^) with increasing workload; and b) a respiratory exchange ratio of at least 1.10.

### Data Analyses

The VO_2peak_ was determined by the highest 30 second average of oxygen uptake (VO_2_ ml·kg^−1^·min^−1^). The VT was determined using the three-method procedure proposed by Gaskill et al. [54]. This involved agreement in the visual determination of VT using; a) ventilatory equivalent method; b) Excess CO_2_ method; and c) modified V-slope method. The RCP was determined accordingly to Beaver et al. [55]. This involved agreement in the visual determination of RCP between plots of a) minute ventilation over CO_2_ production; and b) ventilatory equivalent of CO_2_ over time. The determination of gas exchange thresholds; VT and RCP were agreed upon by two raters. No large differences between the raters were observed. Participants’ mean VT and RCP occurred at 68±3% and 90±2% VO_2peak_, respectively.

A 30 second baseline measure of cerebral haemodynamics (ΔO_2_Hb, ΔHHb, ΔtHb and ΔHbDiff) was recorded prior to exercise. Data were exported every 10 seconds and normalised to express the magnitude of changes from the baseline period (arbitrarily defined as 0 µM) at the start of exercise. A 20 second average of cerebral haemodynamic variables was extracted at time points corresponding to intensities of exercise; 80% of VT (below VT), VT, RCP and end of exercise (End). Corresponding affective responses were selected at time points corresponding to each of the intensities of exercise.

### Statistical Analyses

The independent variables were Region (1–8; dorsal 1, 3, 5 and 7; ventral 2, 4, 6, 8) and Time (corresponding to pre-exercise and intensities below VT, VT, RCP and End). The dependent variables were cerebral haemodynamics (ΔO_2_Hb, ΔHHb, ΔtHb and ΔHbDiff) and affective responses.

To examine hypothesis 1, a series of Region (8; 1–8) by Time (4; below VT, VT, RCP, End) analysis of variance (ANOVA) with repeated measures were conducted for each of the cerebral haemodynamic variables (ΔO_2_Hb, ΔHHb, ΔtHb and ΔHbDiff). To examine hypothesis 2, a one-way ANOVA with repeated measures for Time (5; pre-exercise, and at time points corresponding to below VT, VT, RCP and End) was conducted for affective responses. Finally, to examine hypothesis 3, Pearson’s bivariate correlation analyses were conducted to examine the relationships between cerebral oxygenation (ΔO_2_Hb) and affective responses during exercise. All statistical analysis was performed using SPSS v. 18.0 (IBM Corp., Armonk, NY, USA). Data were assessed for normality using Shapiro-Wilk tests. Greenhouse Geisser corrections were applied if the assumption of sphericity was not met and significant main and interaction effects (*p*<.05) were followed up using repeated measures contrasts. Effect sizes associated with *F* statistics were expressed as eta squared (η^2^) defined as small (.01), medium (.06) and large (.14) [56]. Values are mean ± SD unless otherwise stated.

## Results

Participants’ physiological data (VO_2_) at each time point corresponding to each of the intensities of exercise were: below VT 22.8±2.9; at VT 28.5±3.7; RCP 37.7±4.6; and End 41.8±5.2 ml·kg^−1^·min^−1^. The mean duration of the exercise test was 641±126 seconds.

### Cerebral Haemodynamics (Hypothesis 1)

#### Cerebral ΔO_2_Hb

**Figure 2:**
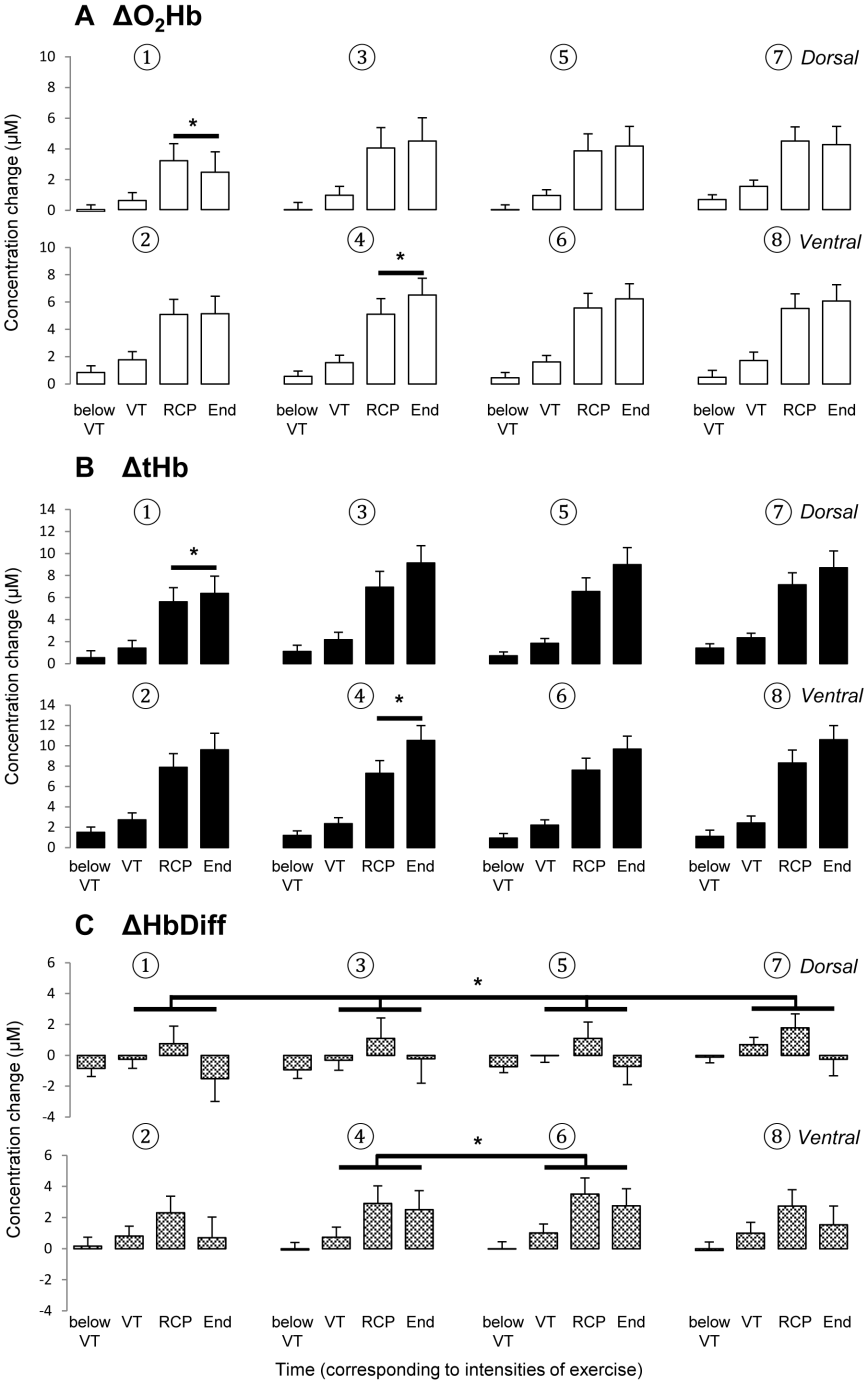
Cerebral haemodynamics during exercise. (A) oxygenation (ΔO_2_Hb), (B) total haemoglobin (ΔtHb), and (C) haemoglobin difference (ΔHbDiff) in sub regions (<$>\raster="rg1"<$> to <$>\raster="rg8"<$>; odd numbers identify dorsal and even numbers identify ventral areas) of the prefrontal cortex at each time point corresponding to below ventilatory threshold (below VT), at VT, respiratory compensation point (RCP) and the end of exercise (End). Mean ± SEM. *indicates significant difference, *p*<.05.

Significant main effects of Region, *F*(4, 105) = 3.67, *p = *.006, η^2^ = .03, and Time, *F*(1, 27) = 16.21, *p*<.001, η^2^ = .27, and a Region by Time, *F*(5, 123) = 4.54, *p*<.001, η^2^ = .01, interaction were recorded for ΔO_2_Hb. Follow up tests indicated that ΔO_2_Hb was greater in Regions 2, 4, 6, 8 (ventral) than Regions 1, 3, 5, 7 (dorsal) (Table 1). Cerebral ΔO_2_Hb increased from below VT (.35±1.65) to VT (1.33±2.17) and VT to RCP (4.60±5.03), but remained stable RCP to End (4.90±5.78). The two factor interaction indicated that from RCP to End, ΔO_2_Hb increased in Region 4 and declined in Region 1 (Figure 2A).

**Table 1:** Oxygenation (ΔO_2_Hb) and haemoglobin difference (ΔHbDiff; µM) by Region (<$>\raster="rg1"<$> to <$>\raster="rg8"<$>).

	Right hemisphere		Left hemisphere		
	<$>\raster="rg1"<$>	<$>\raster="rg3"<$>	<$>\raster="rg5"<$>	<$>\raster="rg7"<$>	
ΔO_2_Hb	1.51±3.86	2.35±4.45	2.22±3.38	2.72±3.09	*Dorsal*
ΔHbDiff	−.46±4.31	−.09±4.83	−.09±3.37	0.53±3.18	
	**<$>\raster="rg2"<$>**	**<$>\raster="rg4"<$>**	**<$>\raster="rg6"<$>**	**<$>\raster="rg8"<$>**	
ΔO_2_Hb*	3.21±3.79	3.43±3.65	3.46±3.33	3.45±3.68	*Ventral*
ΔHbDiff*	0.99±4.31	1.52±3.93	1.82±3.43	1.29±3.86	

Mean ± SD. *indicates significantly greater than other regions, *p*<.05.

#### Cerebral ΔHHb

A significant main effect of Time, *F*(1, 27) = 77.54, *p*<.001, η^2^ = .44, was recorded for ΔHHb. Follow up tests indicated that ΔHHb remained stable from below VT (70±.77) to VT (.87±.76), then increased from VT to RCP (2.57±1.47) and RCP to End (4.30±2.03).

#### Cerebral ΔtHb

A significant main effect of Time, *F*(1, 27) = 33.71, *p<*.001, η^2^ = .42, and a Region by Time, *F*(5, 125) = 2.66, *p = *.024, η^2^ = .01, interaction were recorded for ΔtHb. Follow up tests indicated that ΔtHb increased from below VT (1.06±1.70), to VT (2.19±2.18), RCP (7.17±5.73) and End (9.20±6.64). The two factor interaction indicated that from RCP to End, ΔtHb increased in Region 4 and remained stable in Region 1 (Figure 2B).

#### Cerebral ΔHbDiff

A significant main effect of Region, *F*(4, 91) = 3.28, *p = *.016, η^2^ = .05, and a Region by Time, *F*(5, 120) = 5.39, *p*<.001, η^2^ = .02, interaction were recorded for ΔHbDiff. Follow up tests indicated that ΔHbDiff was greater in Regions 2, 4, 6, 8 (ventral) than Regions 1, 3, 5, 7 (dorsal) (Table 1). The two factor interaction indicated that from VT to RCP and RCP to End, ΔHbDiff increased and was greater in Regions 4 and 6, respectively, than Regions 1, 3, 5 and 7 (dorsal) (Figure 2C).

### Affective Responses (Hypothesis 2)

A significant main effect of Time, *F*(2, 39) = 68.63, *p*<.001, η^2^ = .74, was recorded for affective responses. Affective responses significantly declined from pre-exercise (2.4±1.1) to below VT (1.7±1.0), remained stable until VT (1.5±1.0) and significantly declined from VT to RCP (−.8±1.8) and RCP to End (−2.0±1.8).

### Associations between Cerebral Haemodynamics and Affective Responses (Hypothesis 3)

Below VT and at VT, no significant relationships were shown between affective responses and ΔO_2_Hb in any region of the PFC. However, at RCP, significant inverse relationships were shown between affective responses and ΔO_2_Hb in Regions 1 and 2 in the right hemisphere; and in all Regions (5–8) in the left hemisphere. At End, significant inverse relationships were shown between affective responses and ΔO_2_Hb in Region 4 in the right hemisphere; and in all Regions (5–8) in the left hemisphere (Table 2).

**Table 2:** Associations (*r*) between cerebral oxygenation (ΔO_2_Hb) and affective responses.

	Time (corresponding to intensities of exercise)			
Region	below VT	VT	RCP	End
1	−.17	−.09	−.55**	−.38
2	−.12	−.08	−.53**	−.33
3	−.06	−.08	−.29	−.30
4	−.00	−.11	−.36	−.46*
5	−.30	−.17	−.50*	−.52*
6	−.07	−.09	−.59**	−.56*
7	−.09	−.08	−.62**	−.55*
8	−.04	−.11	−.53**	−.46*

*p<.05, **p<.01.

## Discussion

The present study investigated the functional capacity (activation in 8 sub regions in the left and right hemispheres) of the PFC using multi-channel NIRS during incremental exercise to volitional exhaustion. The pattern of the cerebral haemodynamic and affective responses were examined at intensities of exercise standardised to individual gas exchange thresholds [48], [50], and data were extracted at time points corresponding to below VT, VT, RCP and the end of exercise. In line with the dual-mode model [16], [17], and evidence of cognitive control and lateralized processes, a reduced functional capacity or deregulation may occur in different regions of the PFC during exercise at intensities of exercise above VT, which may explain the regulation of affective responses during exercise.

### Hypothesis 1: Cerebral Haemodynamic Responses

The results support hypothesis 1with differential haemodynamic responses dependent on the level of exercise intensity. At intensities of exercise below VT, deoxygenation (ΔHHb) remained stable whereas oxygenation (ΔO_2_Hb) increased; resulting in an increase in blood volume (ΔtHb) indicating greater activation (increased ΔHbDiff) in all regions of the PFC. The PFC appeared to be adequately supplied with oxygen relative to the demand.

At intensities of exercise at VT, greater oxygenation (ΔO_2_Hb) in bilateral ventral regions (2, 4, 6, 8) were observed. As the intensity of exercise increased from VT to RCP, oxygenation (ΔO_2_Hb) and deoxygenation (ΔHHb) increased in all regions, resulting in an increase in blood volume (ΔtHb) indicating activation (increased ΔHbDiff). However, greater oxygenation (ΔO_2_Hb) and activation (ΔHbDiff) were shown in ventral regions (2, 4, 6, 8) than all dorsal regions (1, 3, 5, 7).

At intensities of exercise from RCP to the end of exercise, oxygenation (ΔO_2_Hb) continued to increase in ventral regions (greatest in region 4 followed by regions 6 and 8), but remained stable or declined in dorsal regions (1, 3, 5, 7). Deoxygenation (ΔHHb) continued to increase in all regions and blood volume (ΔtHb) increased in most regions except the most lateral area in the right dorsolateral region of the PFC (region 1). The increase in blood volume (sum of ΔO_2_Hb and ΔHHb) indicates activation in ventral regions and reduced activation in the dorsal regions at these intensities. This is because the ventral regions showed an increase in oxygenation (ΔO_2_Hb) coupled with an increase in deoxygenation (ΔHHb; therefore adequate oxygen supply for demand; increased ΔHbDiff). However, the dorsal regions showed a decline or plateau in oxygenation (ΔO_2_Hb) coupled with an increased deoxygenation (ΔHHb: therefore lack of oxygen supply to demand; decreased ΔHbDiff). Activation in bilateral ventral regions located closest to the midline (regions 4 and 6; the anterior PFC, approximately BA 10) indicated the greatest activation at the end of exercise. The overall pattern of the cerebral haemodynamic response during incremental exercise is consistent with previous studies [48]; however the different responses between the multiple regions of the PFC measured is a novel finding.

The dual-mode model explains that exercising at intensities below VT would not induce sufficient sensory input from the body to challenge the individual, and therefore the capacity of the PFC. However, as the intensity of exercise increases, sensory input from the body would challenge the capacity of the PFC to maintain activation. According the transient hypofrontality hypothesis [18], [19], the first stage of deregulation would occur in the dorsolateral areas of the PFC and the second stage of deregulation would occur in the ventromedial areas of the PFC. In this study during exercise above the VT, reduced activation was observed in the left and right dorsolateral frontal areas (corresponding to regions 1, 3, 5, 7) and greater activation in ventral regions. As the intensity increased, greater activation was observed in ventral regions (4 and 6) indicating the potential shift in metabolic resources from dorsal regions (regions 1, 3, 5 and 7; stage one) towards ventromedial areas (located beneath regions 4 and 6; stage two). Therefore, during incremental exercise the cerebral haemodynamic response appeared to follow a top-down (dorsal-ventral) direction from VT to RCP, and outside-in (lateral to midline) direction from RCP to the end of exercise.

### Hypothesis 2: Affective Responses

The results support hypothesis 2 with different affective responses dependent on the level of exercise intensity. Despite a decline in affective responses from pre-exercise to intensities below VT, affective responses remained positive until intensities above VT. However, at RCP and the end of exercise affective responses were uniformly negative. This pattern of affective responses is consistent with the dose-response relationship of the intensity of exercise [1]. According to the dual-mode model, sensory input from the body is intensified (above VT) and the functional capacity of the PFC is challenged, presumably due to increased activity in subcortical areas of the brain. Maintained activation in the PFC would serve to maintain a more positive affective response through cognitive control processes. Prior work has shown that maintained PFC activation through the use of a cognitive strategy results in a more positive affective response during exercise at intensities above the VT, but not the RCP [57].

### Hypothesis 3: Associations between Cerebral Haemodynamic and Affective Responses

The results support hypothesis 3 with associations between haemodynamic and affective responses dependent on the level of exercise intensity. No associations between cerebral oxygenation and affective responses were shown at intensities below VT and at VT. From pre-exercise to VT, the PFC appeared to be adequately saturated (sufficient oxygen supply [ΔO_2_Hb] vs. demand [ΔHHb] therefore greater ΔHbDiff) and a positive affective response was reported. It could be expected that at these intensities, PFC activation would be associated with positive affective responses if cognitive control processes influenced the regulation of affective responses. However, the correlations between cerebral oxygenation and affective responses are very weak (see Table 2). It is proposed that at these intensities sensory input from the body is not sufficient enough to induce negative affect and threaten the capacity of the PFC; therefore cognitive control processes may not be actively engaged.

At intensities above VT (i.e. RCP and End), cerebral oxygenation (ΔO_2_Hb) in both the left and right hemispheres was inversely associated with affective responses. In the left hemisphere at intensities of exercise at the RCP and the end of exercise, greater cerebral oxygenation (ΔO_2_Hb) in all regions was associated with a more negative affective response. Greater activation in the left hemisphere in the presence of a negative affective response may reflect increased cognitive control processes towards a less negative/more positive affective response [21], [23].

In the right hemisphere at intensities of exercise at RCP, a more negative affective response was associated with greater cerebral oxygenation in the most lateral (ventral-dorsal) regions (1 and 2), but not those closest to the midline (3 and 4). However, at the end of exercise, only the ventral region closest to the midline (region 4) showed an inverse relationship between cerebral oxygenation (ΔO_2_Hb) and affective responses. This region indicated a significant increase in oxygenation (ΔO_2_Hb) between RCP and the end of exercise. Although some caution should be taken in the interpretation of these moderate linear correlations, the association between oxygenation (ΔO_2_Hb) in both hemispheres, but particularly in the right hemisphere at intensities above VT is novel. Lateralised areas of the PFC have been shown to be involved in the cognitive control of negative affect [24], [27]. Furthermore, relative right hemisphere activation is associated with negative affect [31], [32], which may indicate processes related to active avoidance of negative affect. These findings provide preliminary indications that activation in sub regions of the PFC may be involved in the regulation of negative affective responses during exercise at intensities above VT. It is proposed that increased oxygenation (i.e. blood flow) in sub regions of the PFC is induced by attempts of active cognitive coping with a negative affective stimulus, which would serve to reduce activity in the amygdala [23], [26], [28]–[30].

### Limitations and Future Directions

The interpretation of these results is predominantly speculative for two reasons. Firstly, cognitive control processes proposed to regulate affective responses were not measured during exercise. Secondly, no measures of subcortical areas of the brain (i.e. the amygdala) were recorded. However, this study did measure functional capacity of the PFC (reflected through haemodynamics) to host such cognitive control processes to provide top-down regulation of subcortical areas. An extension of this study would be to employ a cognitive control paradigm to determine the effects upon PFC haemodynamics and affective responses in a control and exercise condition. An understanding of the role of the PFC during exercise will provide some confirmation of a neural basis of the dose-response relationship of affective responses to the intensity of exercise.

The different patterns of the cerebral haemodynamic response in sub regions of the PFC as the intensity of exercise increased may explain some of the variability previously reported. Rooks et al. [48] indicated large variability in cerebral oxygenation (ΔO_2_Hb) at sub maximal (>60% VO_2peak_) and near maximal (VO_2peak_) intensities. The authors attributed some of this variability to the individual’s training status (VO_2peak_), reported by Rooks et al. [48] to have an average VO_2peak_ of 40.3±10.4 ml·kg^−1^·min^−1^ similar to that reported in the present study (41.8±5.2 ml ·kg^−1^·min^−1^). The participants in the current study were all physically active however no measure of training status was assessed. As recommended by the authors, the intensities of exercise in this study were standardized to individual metabolic markers. Using multi-channel NIRS, altered patterns of the cerebral haemodynamic response were observed in the different sub regions measured (i.e. dorsal regions indicate less activation [reduced ΔO_2_Hb] than ventral regions) and between hemispheres (i.e. greater ΔO_2_Hb in region 4) at intensities above VT (68% VO_2peak_). These findings indicate that variability in the cerebral haemodynamic response may be due to the regions (i.e. the placement of optodes) from which the haemodynamic response is measured. This was not considered by Rooks et al. [48]. Although, the authors did examine the distance between optodes (< or >4 cm), no effect upon the cerebral haemodynamic response was indicated. Prior studies examining the influence of cerebral oxygenation during incremental exercise have measured cerebral and muscle oxygenation under conditions such as hypoxia [49] and hyperoxia [58]. Measures of cognitive and affective processes in these types of studies are warranted to encompass additional factors associated with limited cerebral oxygenation during exercise, aside from those related to motor control. Future research, should consider methodological limitations associated with using NIRS during exercise such as approximate optode placement (within-between subjects and sessions) and sampling procedures as highlighted previously [46], [49] and population characteristics (i.e. fitness and training status). Although, the spatial resolution of NIRS is poor compared to other neuroimaging techniques (such as magnetic resonance imaging), NIRS is less influenced by movement artifact making it suitable for use during exercise.

## Conclusion

The findings of the current study indicate the potentially limited functional capacity of the PFC during exercise at intensities above the VT. Some support for the dual-mode model as a potential neural basis of the dose-response effects of the intensity of exercise and affective responses has been provided. The study has indicated that prefrontal haemodynamics (which reflect functional activation) during exercise shifts in a top-down manner from dorsal to ventral regions, with greatest activation in anterior regions (closest to the midline) near exhaustion. In the left hemisphere, consistent inverse associations between affective responses and oxygenation in all (dorsal and ventral) regions were observed at intensities above the VT. In the right hemisphere, inverse associations between affective responses and oxygenation in the lateral (dorsal and ventral) and the midline (ventral) regions were observed at intensities at the RCP and the end of exercise, respectively.
